# A Pilot Study of 24-h Motor Activity Patterns in Multiple Sclerosis: Pre-Planned Follow-Up at 2 Years

**DOI:** 10.3390/clockssleep3030023

**Published:** 2021-06-28

**Authors:** Lorenzo Tonetti, Federico Camilli, Sara Giovagnoli, Vincenzo Natale, Alessandra Lugaresi

**Affiliations:** 1 Department of Psychology “Renzo Canestrari”, University of Bologna, Viale Berti Pichat 5, 40127 Bologna, Italy; sara.giovagnoli@unibo.it (S.G.); vincenzo.natale@unibo.it (V.N.); 2 Dipartimento di Scienze Biomediche e Neuromotorie, Università di Bologna, 40139 Bologna, Italy; federico.camilli@studio.unibo.it (F.C.); alessandra.lugaresi2@unibo.it (A.L.); 3 IRCCS Istituto delle Scienze Neurologiche di Bologna, UOSI Riabilitazione Sclerosi Multipla, 40139 Bologna, Italy

**Keywords:** multiple sclerosis, actigraphy, prospective study, 24-h motor activity pattern, prognosis, clinical course

## Abstract

Early multiple sclerosis (MS) predictive markers of disease activity/prognosis have been proposed but are not universally accepted. Aim of this pilot prospective study is to verify whether a peculiar hyperactivity, observed at baseline (T0) in early relapsing-remitting (RR) MS patients, could represent a further prognostic marker. Here we report results collected at T0 and at a 24-month follow-up (T1). Eighteen RRMS patients (11 females, median Expanded Disability Status Scale-EDSS score 1.25, range EDSS score 0–2) were monitored at T0 (mean age 32.33 ± 7.51) and T1 (median EDSS score 1.5, range EDSS score 0–2.5). Patients were grouped into two groups: responders (R, 14 patients) and non-responders (NR, 4 patients) to treatment at T1. Each patient wore an actigraph for one week to record the 24-h motor activity pattern. At T0, NR presented significantly lower motor activity than R between around 9:00 and 13:00. At T1, NR were characterized by significantly lower motor activity than R between around 12:00 and 17:00. Overall, these data suggest that through the 24-h motor activity pattern, we can fairly segregate at T0 patients who will show a therapeutic failure, possibly related to a more active disease, at T1. These patients are characterized by a reduced morning level of motor activation. Further studies on larger populations are needed to confirm these preliminary findings.

## 1. Introduction

Multiple sclerosis (MS) is the most frequent demyelinating disease of the central nervous system (CNS) and one of the main causes of disability in young adults worldwide. MS is a chronic disease due to an inflammatory immune-mediated process against the white matter, leading to axonal degeneration. It is well-known that MS symptomatology highly differs according to the specific area of the CNS affected [1].

MS has a prevalence of 50–300 per 100,000 people, affecting between two and three million people worldwide [2]. Sex differences in MS prevalence have been consistently reported, with a sex ratio close to 3F:1M in most of the developed countries [3]. The onset of MS usually occurs between the age of 20 and 40 although an increased rate of MS has been recently observed in pediatric subjects as well as in older (>60 years of age) adults [4].

Different types of MS course can be differentiated: relapsing-remitting MS (RRMS); secondary progressive MS (SPMS); primary progressive MS (PPMS). While RRMS is the most common form at onset (i.e., around 85–90% of patients), along the disease course some patients experience progressive worsening over time, leading to a shift to SPMS. Although some early prognostic indicators have been identified, at onset, the future disease course is quite unpredictable. Therefore, the identification of further prognostic/predictive markers of disease activity or progression would be of utmost importance.

In recent years, a potential involvement of the circadian timing system in the multifactorial etiopathology of MS has been pointed out by a few studies. In particular, Lavtar and colleagues [5] have investigated the relationship between genetic variability of two genes, *ARNTL* and *CLOCK*, key regulators of circadian rhythms, and the risk of MS. They interestingly pointed out that *ARNTL* rs3789327 CC and *CLOCK* rs6811520 genotypes were associated with a higher risk of MS. A more recent study, carried out by Gasperoni and colleagues [6], developed a theoretical framework of MS etiopathogenesis showing that people living in countries presenting a circadian disruptive factor, namely the daylight-saving time (DST), reported a 6.35 times higher prevalence of MS compared to individuals living in countries at the same latitude but that did not adopt the DST. Overall, the studies briefly reviewed seem to support the potential involvement of circadian factors in the multifactorial etiopathology of MS.

Based on these findings, we recently chose to perform a study aimed at verifying whether the 24-h motor activity pattern, recorded through actigraphy, of 35 early RRMS patients (less than 24 months from the diagnosis), presented specific alterations through a comparison with the 24-h motor activity pattern of 35 healthy controls matched for sex and age [7]. We chose to examine the 24-h motor activity pattern, through a statistical framework specifically developed for the analysis of actigraphic data (functional linear modeling, FLM) [8], because a previous study [9], examining the 24-h motor activity pattern through FLM, detected a potential trait marker of a different disease, namely the attention-deficit hyperactivity disorder. The results of our actigraphic study in early RRMS patients highlighted a peculiar hyperactivity in patients at 05:00 that was interpreted as a sign of a hyperactive hypothalamus-pituitary-adrenal axis and the consequent increase in the release of cortisol.

To further investigate the significance of our findings, we chose to implement a prospective study with a periodic pre-planned follow-up, every 24 months, over an overall time span of 10 years. The aim of this prospective study is to verify if such peculiar hyperactivity, observed in early RRMS patients, may be associated with a different disease course, potentially representing a prognostic/predictive motor marker of disease progression.

In the current paper, we report the preliminary results of the first follow-up (time 1, T1) assessment on MS patients who were actigraphically monitored two years earlier at baseline (T0) assessment. Because at T1 all patients still presented the RR type of MS, we considered therapy escalation as a marker of a worse disability trajectory, i.e., as a prognostic surrogate, grounded on the presence of disease activity that did not adequately respond to first line treatments [10,11,12]. Accordingly, at T1 the group of patients was split into two sub-samples based on the treatment response: responders (showing a satisfactory response to treatment, i.e., therapeutic success, responder group, R) and non-responders (showing disease activity requiring a switch to second line treatment, i.e., therapeutic failure, non-responder group, NR).

## 2. Results

### 2.1. Between-Subjects Comparison at T0 between the Non-Responder (NR) Group and the Responder (R) Group

The distribution of males and females among the R and NR groups was not significantly different (χ^2^_1_ = 0.27; *p* = 0.61). Moreover, the R (34 ± 7.15 years) and NR (35.25 ± 9.43 years) groups did not differ significantly in age (t_16_ = 0.29; *p* = 0.78; Cohen’s d = 0.16). Keeping in mind the significance level corrected through Bonferroni, the EDSS score of R (1.18 ± 0.50) was not significantly different (t_16_ = 2.63; *p* = 0.02; Cohen’s d = 1.51) from the EDSS score of NR (1.88 ± 0.25).

#### 2.1.1. Actigraphic Parameters

As shown in Table 1, no significant differences in actigraphic sleep/wake measures were observed between groups.

#### 2.1.2. 24-h Motor Activity Pattern

**Figure 1:**
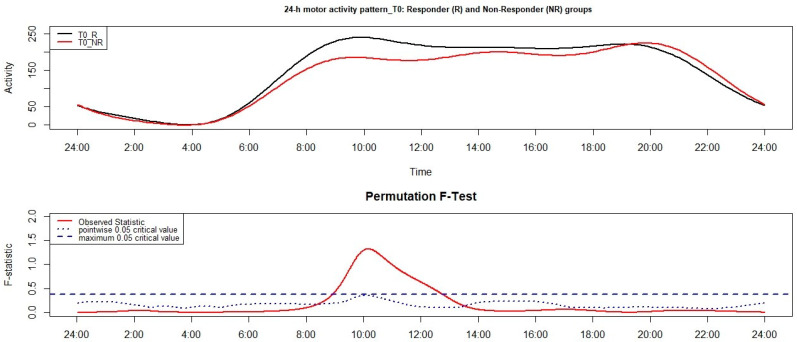
Results of the functional linear modeling applied to the group comparison between responder (R) and non-responder (NR) groups at T0. The functional forms of the 24-h motor activity patterns of groups are reported in the upper panel while the results of the non-parametric permutation F-test in the lower panel. Significant results are observed when the solid red line is above the blue dashed line.

Figure 1 shows the results of the FLM applied to the comparison of the 24-h motor activity pattern between the R and NR group. Significant differences were observed between around 9:00 and 13:00, with higher motor activity in the R group.

### 2.2. Between-Subjects Comparison at T1 between the NR Group and the R Group

NR (1.63 ± 0.48) and R (1.39 ± 0.66) did not significantly differ in the EDSS score (t_16_ = 0.65; *p* = 0.52; Cohen’s d = 0.38). Moreover, the number of relapses was not significantly different (t_16_ = 1.72; *p* = 0.10; Cohen’s d = 0.97) in NR (0.75 ± 0.5) compared to R (0.29 ± 0.47).

#### 2.2.1. Actigraphic Parameters

Table 2 shows the comparison between NR and R group with reference to each actigraphic sleep/wake parameter measured at T1. We did not observe any significant differences between groups.

#### 2.2.2. 24-h Motor Activity Pattern

**Figure 2:**
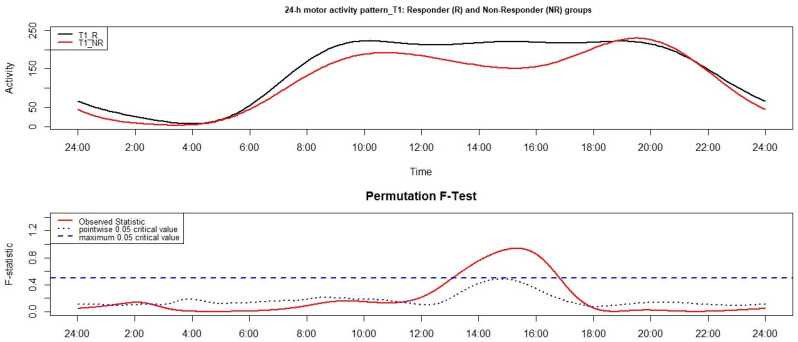
Results of the functional linear modeling applied to the group comparison between responder (R) and non-responder (NR) groups, monitored at T1. The functional forms of the 24-h motor activity patterns of groups are reported in the upper panel while the results of the non-parametric permutation F-test in the lower panel. Significant results are observed when the solid red line is above the blue dashed line.

The comparison, through functional linear modeling (FLM), between the 24-h motor activity pattern of NR and R group, monitored at T1, reached statistical significance between around 13:00 and 17:00 with higher motor activity in the R group (Figure 2).

### 2.3. R Group: Within-Subject Comparison between T0 and T1

#### 2.3.1. Actigraphic Parameters

Comparing the actigraphic sleep/wake parameters measured at T0 and T1 in the responder (R) group, no significant differences were observed (Table S1).

#### 2.3.2. 24-h Motor Activity Pattern

No significant differences between the 24-h motor activity pattern of the R group recorded at T0 and T1 have been found (see Figure S1).

### 2.4. NR Group: Within-Subject Comparison between T0 and T1

#### 2.4.1. Actigraphic Parameters

Performing a set of dependent samples t-tests, in order to compare each actigraphic sleep/wake measure obtained at T0 and T1 within the NR group, we did not observe any significant differences (Table S2).

#### 2.4.2. 24-h Motor Activity Pattern

We observed significant differences between the 24-h motor activity pattern monitored at T0 and T1 in the NR group of patients (Figure S2). In particular, at T1 patients moved significantly less than at T0 between around 15:00 and 16:00.

## 3. Discussion

The main general aim of the current prospective study was to determine whether the peculiar hyperactivity, previously reported in newly diagnosed patients with early RRMS [7], was related to a different disease course. Therefore, the main question we were trying to answer with the current study was: can this peculiar hyperactivity represent a prognostic/predictive motor marker of disease progression?

To this end, in the current article we chose to present the results of the comparison between 24-h motor activity pattern (besides actigraphic sleep/wake cycle) of MS patients monitored at T0 and two years later, that is at T1.

To sum up the main findings, as regards the actigraphic sleep/wake cycle measures, we did not observe any significant differences neither in the between-group nor in the within-group comparisons. Although not significant, because of the Bonferroni correction, the results on the actigraphic sleep/wake cycle measures, reported in Table 1 and Table 2, are potentially interesting. Indeed, NR and R mainly differ in the sleep maintenance, as observed through the WASO and AWK > 5 parameters. The difference in the minutes of WASO between NR and R groups increased by 13% at T1 compared to T0, while the difference in the number of AWK > 5 between NR and R groups increased by 14% at T1 compared to T0. This pattern of results seems to point out that the difference between NR and R groups in sleep maintenance is more marked at T1 compared to T0, as if an impaired sleep became overt later than the alterations in the 24-h motor activity pattern.

With reference to the results on the 24-h motor activity pattern, significant differences were observed at both between-group comparisons and at one out of two within-group comparisons. More in detail, at T0, the NR group presented significantly lower motor activity than the R group in a morning time window, namely between around 9:00 and 13:00; the strongest difference between groups was observed around 10:00. At T1, the NR group was characterized by significantly lower motor activity than the R group between around 13:00 and 17:00, with the highest difference around 16:00. While we did not observe any significant differences between 24-h motor activity pattern of the R group monitored at T0 and T1, the NR group showed a significantly reduced motor activity at T1 compared to T0 between 15:00 and 16:00, with the strongest difference being observed around 16:00.

It is extremely interesting that, already at T0, the NR group of patients (those who two years later will have switched to a second line treatment due to inefficacy of first line treatments) was characterized by a markedly lower motor activity during the morning. A definite explanation is not yet possible. However, keeping in mind that fatigue, an early characteristic feature of MS, can be disabling also for patients with low disability as assessed through the Expanded Disability Status Scale (i.e., range 0–2 at T0) [13], it is conceivable that this activity pattern may be associated to a sort of energy-saving strategy [14] unintentionally adopted by patients with more active disease, who will not respond satisfactorily to MS first line treatments. The trend observed toward a higher EDSS score in the NR is in line with this hypothesis.

Moreover, our data can be conceived as somewhat in line with those previously reported by Motl and colleagues in 2012 [15] highlighting a relationship between higher subjectively reported premorbid physical activity and slower disability progression of RRMS over time.

Another interesting observation comes from the similarity of the results observed at both between-group comparison at T1 (NR vs. R groups) and at the within-group comparison (T0 vs. T1) in the NR group, i.e., a markedly lower motor activity in the NR group compared to the R group and at T1 compared to T0, respectively, in the afternoon with the highest statistical significance observed around 16:00. Overall, it is possible to hypothesize that the NR group, between T0 and T1, presented a worsening of the degree of demyelination which, in turn, could potentially explain the significantly lower motor activity at a specific time of the day, when core body temperature increases [16], leading to an impaired conduction in demyelinated fibers. Indeed, it has been previously reported that temperature sensitivity in MS (a very common condition characterized by temporary worsening of neurological symptoms due to the increase of body temperature) is mainly explained by the changes in core body temperature leading to a slowing down or interruption of neural conduction [17]. It is well-known that an increased core temperature is related to motor symptoms, as fatigue, that could translate into a reduced motor activity [17].

Hopefully, the continuation of the current prospective study will allow to understand the trajectories of the disease course in these MS patients. It would be of utmost importance to understand whether the reduced morning motor activity, already detected at T0 in those who at T1 will show a therapeutic failure, is related, over the years, to a more severe MS course, and might therefore represent a motor prognostic-predictive marker. Furthermore, in case such trajectories of disease course should be disclosed, it will be necessary to provide normative data of motor activity, currently missing, to detect the presence of an altered 24-h motor activity pattern.

The present study is not free from limitations. First, since no conversion to SPMS occurred between T0 and T1, we considered therapy escalation as prognostic surrogate indicating a more aggressive course. Second, the sample size of the whole group and of the R and NR subgroups of patients, identified through the implementation of such prognostic surrogate, is small. Third, although not significant, the EDSS score at T0 was higher in NR compared to R; we wish to underline that the range of the EDSS score at T0 was 0–2, indicating a low disability, in both groups.

## 4. Methods

### 4.1. Participants

A cohort of 21 early (less than 24 months from the diagnosis) RRMS patients (14 females and 7 males) was actigraphically monitored at baseline (T0). Mean age was 32.05 ± 7.83 years. Females (33.5 ± 7.97 years) and males (29.14 ± 7.22 years) did not significantly differ in age (t_19_ = 1.22; *p* = 24; Cohen’s d = 0.56). The mean EDSS score was 1.33 ± 0.54.

Patients were eligible for enrolment if the following inclusion criteria were verified.

For the first phase: (1) definite diagnosis of MS according to the current diagnostic criteria [2] and MAGNIMS recommendations [18,19,20,21]; (2) age between 18 and 64 years; (3) disease duration <2 years; (4) disease-modifying treatment for no less than 6 months.

For both phases: unchanged symptomatic therapy in the two weeks preceding and for the whole duration of each actigraphic assessment.

For phase 2: same disease-modifying treatment since least 6 months.

At the pre-planned follow-up (time 1, T1), it was not possible to monitor 3 out of 21 patients recruited at T0, due to the first wave of the coronavirus disease (COVID-19) pandemic.

Therefore, overall, 18 MS patients—11 females and 7 males—participated in T1 assessment. The mean age of the overall sample was 34.28 ± 7.42 years. The females’ mean age (36.27±7.14) was not significantly different from males’ mean age (31.14 ± 7.22; t_16_ = −1.48; *p* = 0.16; Cohen’s d = 0.72).

During the clinical interview preceding T1 assessment, factors potentially able to interfere with physiological sleep/wake cycle were verified: (1) relapse or steroid treatment in the last 30 days; (2) diagnosed sleep disorders; (3) history of acute severe pathologies or psychiatric disorders; (4) use of psychoactive drugs; (5) disabilities limiting motor activity; (6) night shift work during the actigraphic monitoring.

At T1, all patients still presented a diagnosis of RRMS. Therefore, to detect an early prognostic/predictive motor marker of the disease course, the whole sample of patients was split into two sub-samples based on the transition (or not) to second-line therapy.

The use of therapy escalation as prognostic surrogate is justified by the fact that second line treatment, in Italy, can only be prescribed in the presence of active disease, not manageable with first line treatments [10,11,12].

Accordingly, two groups of patients were identified, i.e., the responder (R; therapeutic success) and non-responder (NR; therapeutic failure) groups. We defined as responders patients who were clinically stable—no clinically relevant EDSS increase, no relapses during the study period, minimal or no MRI activity (0–1 cumulative unique active lesions)—therefore continuing with the same treatment strategy. We defined as non-responders the remaining patients that switched to a different treatment option (usually a second line drug, such as fingolimod, natalizumab, etc.).

The R group was composed of 14 patients (9 females and 5 males), while the NR group of 4 patients (2 females and 2 males). With reference to each patient of the NR group, we observed the following DMT switches between T0 and T1: (1) from natalizumab to ocrelizumab; (2) from dimethyl fumarate to ocrelizumab; (3) from glatiramer acetate to natalizumab; (4) from fingolimod to ocrelizumab.

### 4.2. Actigraphy

In the present study, we used the actigraph Micro Motionlogger Watch (Ambulatory Monitoring, Inc., Ardsley, NY, USA). The hardware consists of a piezoelectric accelerometer with a sensitivity ≥ 0.01 g. The sampling frequency was 10 Hz while filters were set to 2–3 Hz. We initialized the actigraphs, through the Motionlogger Watchware software (Ambulatory Monitoring, Inc., Ardsley, NY, USA), to collect data, in zero crossing mode, in 1-min epochs. The primary actigraphic output, i.e., motor activity counts, can be converted into a dichotomous variable, i.e., sleep/wakefulness, according to the algorithm, previously validated against polysomnography, by Cole and colleagues [22,23].

### 4.3. Actigraphic Sleep/Wake Measures

To compute some of the most commonly used sleep/wake measures, the definition of which can be found elsewhere [7], we examined the actigraphic files through the Action W 2.7.1150 software (Ambulatory Monitoring, Inc., Ardsley, NY, USA).

As regards sleep, we computed the following parameters: bedtime (BT), get-up time (GUT); time in bed (TIB); midpoint of sleep (MS); sleep motor activity (SMA); sleep onset latency (SOL); total sleep time (TST); wake after sleep onset (WASO); sleep efficiency (SE); awakenings (AWK); awakenings lasting more than 5 min (AWK > 5).

With reference to wake, the following measures were computed: diurnal motor activity (DMA); diurnal total sleep time (DTST); diurnal sleep episodes (NAP); duration of the longest nap (NAPD).

For each patient, the mean of the actigraphic sleep/wake measures of working days only was computed.

### 4.4. 24-h Motor Activity Pattern

For each working day, we extracted the raw motor activity counts, minute-by-minute over the 24 h, through the Action 4 software (Ambulatory Monitoring, Inc., Ardsley, NY, USA). Then, for each patient, we computed the mean profile of raw 24-h motor activity pattern.

### 4.5. Procedure

Patients were requested to wear the actigraph around the wrist of the non-dominant hand 24-h per day for 7 consecutive days. Patients were also requested to push the event-marker button, placed on the top of the actigraph, to signal BT and GUT, allowing the scorer to correctly set the TIB for actigraphic analyses. If patients failed to press the event-marked button at those two times of day, the scorer referred to the replies to the BT and GUT questions of the Italian version [24] of the Core Consensus Sleep Diary [25], daily filled in by patients during the actigraphic recording week. To be included in the research project, each participant provided a written informed consent. The research protocol was approved by the Bologna-Imola Ethics Committee (general protocol number 0122151 of 18 October 2017; study number 17113).

### 4.6. Statistical Analyses

We chose to carry out four comparisons, two between-subjects and two within-subjects.

With reference to the between-subjects comparisons, we compared the R and NR groups at T0 and T1.

As regards the within-subject comparisons, separately for R and NR, we compared data recorded at T0 with those at T1.

With reference to the between-subjects comparisons, we compared the actigraphic sleep/wake measures of R and NR groups through a set of independent samples t-tests using group (two levels, R and NR) as independent variable and the actigraphic measure as dependent variable. As regards the 24-h motor activity pattern, the functional linear modeling (FLM) [8], a statistical framework specifically developed to the analysis of actigraphic data, was used in order to detect if and when the 24-h motor activity patterns of groups statistically differed over the 24 h.

As regards the within-subject comparisons, separately for R and NR, we carried out a set of dependent samples t-tests to compare each actigraphic sleep/wake measure at T0 and T1. Moreover, the 24-h motor activity patterns monitored at T0 and T1 were compared through the FLM.

Because we performed multiple comparisons (*n* = 60), the significance level was corrected through Bonferroni (i.e., 0.05/60) that led to consider as significant, at both independent and dependent samples t-tests, *p*-values less than 0.0008.

## 5. Conclusions

The main take-home message of the current study is that, already in the earliest phases of the disease (i.e., T0), patients who two years later (i.e., T1) will present a therapeutic failure are characterized by lower morning motor activity compared to patients who will present a therapeutic success. Nevertheless, being aware of the limitations of the present study, we wish to underline that the current results must be confirmed by upcoming data. More in detail, it would be extremely interesting if the next waves of the current prospective study will prove the existence of different trajectories of disease course based on the lower morning motor activity recorded at T0, i.e., whether this peculiar, reduced morning activation may represent a prognostic/predictive motor marker of disease course.

## Figures and Tables

**Table 1:** Means and standard deviations of actigraphic sleep/wake measures, measured at T0, in the non-responder (NR) group and responder (R) group.

	NR	R	Statistics		
			t_(16)_	*p* ^a^	Cohen’s d
**Sleep**					
BT	23:32 ± 0:31	23:13 ± 1:07	0.55	0.59	0.31
GUT	07:26 ± 0:58	06:47 ± 0:52	1.31	0.21	0.73
TIB	470.1 ± 64.36	455.1 ± 60.06	0.43	0.67	0.25
MS	03:31 ± 0:34	02:59 ± 0:52	1.15	0.27	0.65
SMA	14.85 ± 6.95	12.44 ± 3.77	0.94	0.36	0.53
SOL	10.75 ± 4.97	13.3 ± 7.22	−0.66	0.52	0.37
TST	416.1 ± 31.60	424.16 ± 57.27	−0.27	0.79	0.15
WASO	42.25 ± 39.70	18.57 ± 9.98	2.15	0.05	1.22
SE	89.32 ± 7.66	93.16 ± 2.87	−1.61	0.13	0.91
AWK	12.05 ± 8.31	9.44 ± 3.93	0.91	0.38	0.52
AWK > 5	3.9 ± 3.39	1.91 ± 0.76	2.16	0.05	0.52
**Wake**					
DMA	191.93 ± 15.46	215.04 ± 23.66	−1.82	0.09	1.03
DTST	34.06 ± 37.19	34.45 ± 40.98	−0.02	0.99	0.01
NAP	4 ± 4.04	4.07 ± 3.38	−0.04	0.97	0.02
NAPD	19 ± 15.27	18.64 ± 21.29	0.03	0.98	0.02

Note: BT = bedtime (h:min); GUT = get-up time (h:min); TIB = time in bed (min.); MS = midpoint of sleep (h:min); SMA = sleep motor activity (counts); SOL = sleep onset latency (min.); TST = total sleep time (min.); WASO = wake after sleep onset (min.); SE = sleep efficiency (%); AWK = awakenings (number); AWK > 5 = awakenings lasting more than 5 min (number); DMA = diurnal motor activity (counts); DTST = diurnal total sleep time (min.); NAP = diurnal sleep episodes (number); NAPD = duration of the longest sleep episode (min.). ^a^ Since multiple comparisons were performed, the Bonferroni correction was applied, leading to consider as significant *p*-values less than 0.0008.

**Table 2:** Means and standard deviations of actigraphic sleep/wake parameters, measured at T1, in the non-responder (NR) group and responder (R) group.

	NR	R	Statistics		
			t_(16)_	*p* ^a^	Cohen’s d
**Sleep**					
BT	23:04 ± 0:39	23:34 ± 1:28	−0.65	0.52	0.37
GUT	07:19 ± 0:40	06:58 ± 1:19	0.51	0.61	0.29
TIB	500.35 ± 54.76	444.04 ± 51.38	1.91	0.07	1.08
MS	03:08 ± 0:24	03:16 ± 1:19	−0.17	0.87	0.10
SMA	16.24 ± 5.70	12.86 ± 3.69	1.44	0.17	0.82
SOL	19.35 ± 11.50	14.78 ± 6.73	1.03	0.32	0.58
TST	434.7 ± 31.55	409.68 ± 52.55	0.90	0.38	0.51
WASO	45.95 ± 31.67	19.69 ± 9.96	2.83	0.01	1.60
SE	90.83 ± 6.16	95.34 ± 2.44	−2.30	0.03	1.31
AWK	12.1 ± 5.34	9.48 ± 3.28	1.23	0.24	0.70
AWK > 5	4.6 ± 2.48	2.33 ± 0.98	2.88	0.01	1.63
**Wake**					
DMA	184.59 ± 12.55	211.52 ± 23.93	−2.14	0.05	1.21
DTST	40.21 ± 34.96	28.40 ± 27.75	0.71	0.49	0.40
NAP	3.46 ± 3.15	3.83 ± 2.63	−0.24	0.81	0.14
NAPD	22.79 ± 16.67	13.05 ± 11.61	1.35	0.20	0.77

Note: BT = bedtime (h:min); GUT = get-up time (h:min); TIB = time in bed (min.); MS = midpoint of sleep (h:min); SMA = sleep motor activity (counts); SOL = sleep onset latency (min.); TST = total sleep time (min.); WASO = wake after sleep onset (min.); SE = sleep efficiency (%); AWK = awakenings (number); AWK>5 = awakenings lasting more than 5 min (number); DMA = diurnal motor activity (counts); DTST = diurnal total sleep time (min.); NAP = diurnal sleep episodes (number); NAPD = duration of the longest sleep episode (min.). ^a^ Since multiple comparisons were performed, the Bonferroni correction was applied, leading to consider as significant *p*-values less than 0.0008.

## Data Availability

The data are not publicly available and cannot be shared due to ethical issue.

## Supplementary Materials

The following are available online at https://www.mdpi.com/article/10.3390/clockssleep3030023/s1. Two tables (i.e., Tables S1 and S2) and two figures (i.e., Figures S1 and S2) are published online alongside the manuscript.
