# Supplementary material for: A Pilot Study of 24-h Motor Activity Patterns in Multiple Sclerosis: Pre-Planned Follow-Up at 2 Years
Source: Clocks Sleep. 2021 Jun 28;3(3):366–76. doi: 10.3390/clockssleep3030023 (PMC8293228; doi:10.3390/clockssleep3030023)
Supplement: Supplementary file 1 [file clockssleep-03-00023-s001.zip › Supplementary material_clockssleep-1249407_25-06-21.pdf]

Table S1. Means and standard deviations of actigraphic sleep/wake parameters measured at T0 and T1 in the responder (R) group.

	T0	T1	Statistics		
			$t_{(13)}$	p^a	Cohen's d
**Sleep**					
BT	23:13 ± 1:07	23:34 ± 1:28	-1.65	.12	.44
GUT	06:47 ± 0:52	06:58 ± 1:19	-.69	.50	.18
TIB	455.1 ± 60.06	444.04 ± 51.38	1.23	.24	.33
MS	02:59 ± 0:52	03:16 ± 1:19	-1.19	.26	.32
SMA	12.44 ± 3.77	12.86 ± 3.69	-.43	.68	.11
SOL	13.3 ± 7.22	14.78 ± 6.73	-.59	.56	.16
TST	424.16 ± 57.27	409.68 ± 52.55	1.64	.13	.44
WASO	18.57 ± 9.98	19.69 ± 9.96	-.34	.74	.09
SE	93.16 ± 2.87	95.34 ± 2.44	-2.84	.01	.76
AWK	9.44 ± 3.93	9.48 ± 3.28	-.03	.98	.01
AWK>5	1.91 ± .76	2.33 ± .98	-1.31	.21	.35
**Wake**					
DMA	215.04 ± 23.66	211.52 ± 23.93	.94	.37	.25
DTST	34.45 ± 40.98	28.40 ± 27.75	.60	.56	.16
NAP	4.07 ± 3.38	3.83 ± 2.63	.34	.74	.09
NAPD	18.64 ± 21.29	13.05 ± 11.61	.97	.35	.26

Note: BT=bedtime (h:min); GUT=get-up time (h:min); TIB=time in bed (min.); MS=midpoint of sleep (h:min); SMA=sleep motor activity (counts); SOL=sleep onset latency (min.); TST=total sleep time (min.); WASO=wake after sleep onset (min.); SE=sleep efficiency (%); AWK=awakenings (number); AWK>5=awakenings lasting more than 5 minutes (number); DMA=diurnal motor activity (counts); DTST=diurnal total sleep time (min.); NAP=diurnal sleep episodes (number); NAPD=duration of the longest sleep episode (min.).

[a] Since multiple comparisons were performed, the Bonferroni correction was applied, leading to consider as significant p-values less than .0008.

Table S2. Means and standard deviations of actigraphic sleep/wake parameters measured at T0 and T1 in the non-responder (NR) group.

	T0	T1	Statistics		
			$t_{(3)}$	p[a]	Cohen's d
**Sleep**					
BT	23:32 ± 0:31	23:04 ± 0:39	2.23	.11	1.13
GUT	07:26 ± 0:58	07:19 ± 0:40	.31	.78	.15
TIB	470.1 ± 64.36	500.35 ± 54.76	-.97	.40	.48
MS	03:31 ± 0:34	03:08 ± 0:24	2.52	.09	1.28
SMA	14.85 ± 6.95	16.24 ± 5.70	-.89	.44	.45
SOL	10.75 ± 4.97	19.35 ± 11.50	-1.38	.26	.69
TST	416.1 ± 31.60	434.7 ± 31.55	-1.05	.37	.53
WASO	42.25 ± 39.70	45.95 ± 31.67	-.25	.82	.12
SE	89.32 ± 7.66	90.83 ± 6.16	-.58	.60	.29
AWK	12.05 ± 8.31	12.1 ± 5.34	-.02	.98	.01
AWK>5	3.9 ± 3.39	4.6 ± 2.48	-.58	.60	.29
**Wake**					
DMA	191.93 ± 15.46	184.59 ± 12.55	.93	.42	.47
DTST	34.06 ± 37.19	40.21 ± 34.96	-1.74	.18	.82
NAP	4 ± 4.04	3.46 ± 3.15	.62	.58	.31
NAPD	19 ± 15.27	22.79 ± 16.67	-.85	.46	.43

Note: BT=bedtime (h:min); GUT=get-up time (h:min); TIB=time in bed (min.); MS=midpoint of sleep (h:min); SMA=sleep motor activity (counts); SOL=sleep onset latency (min.); TST=total sleep time (min.); WASO=wake after sleep onset (min.); SE=sleep efficiency (%); AWK=awakenings (number); AWK>5=awakenings lasting more than 5 minutes (number); DMA=diurnal motor activity (counts); DTST=diurnal total sleep time (min.); NAP=diurnal sleep episodes (number); NAPD=duration of the longest sleep episode (min.).

[a] Since multiple comparisons were performed, the Bonferroni correction was applied, leading to consider as significant p-values less than .0008.

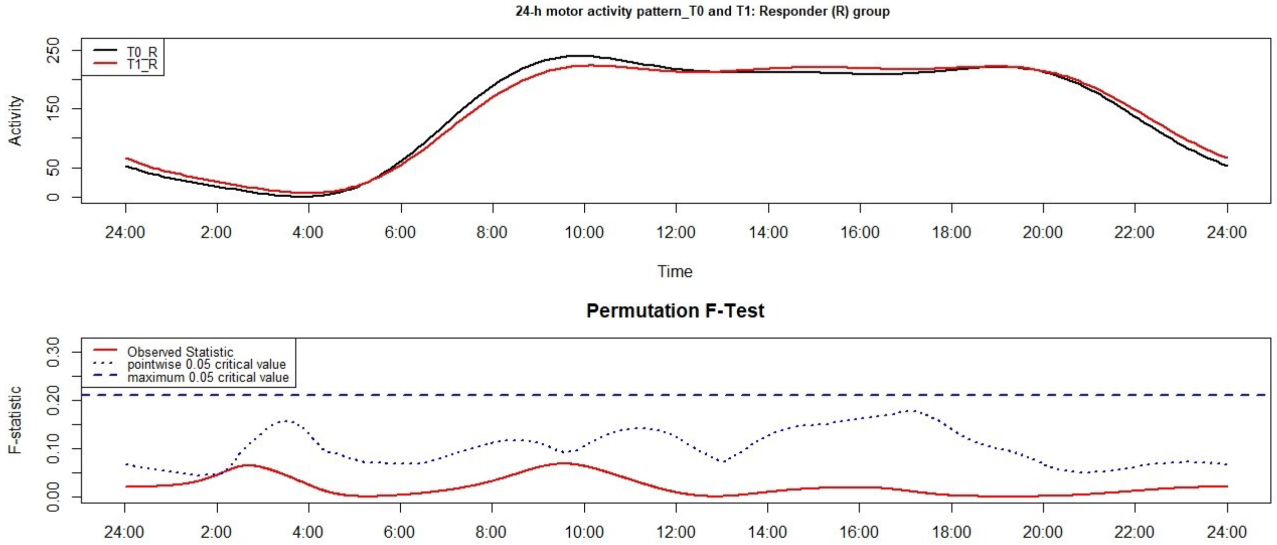

Figure S1 – Results of the functional linear modeling applied to the comparison of 24-h motor activity pattern, monitored at T0 and T1, of the responder (R) group. The functional forms of the 24-h motor activity patterns at T0 and T1 are reported in the upper panel while the results of the non-parametric permutation F-test in the lower panel. Significant results are observed when the solid red line is above the blue dashed line.

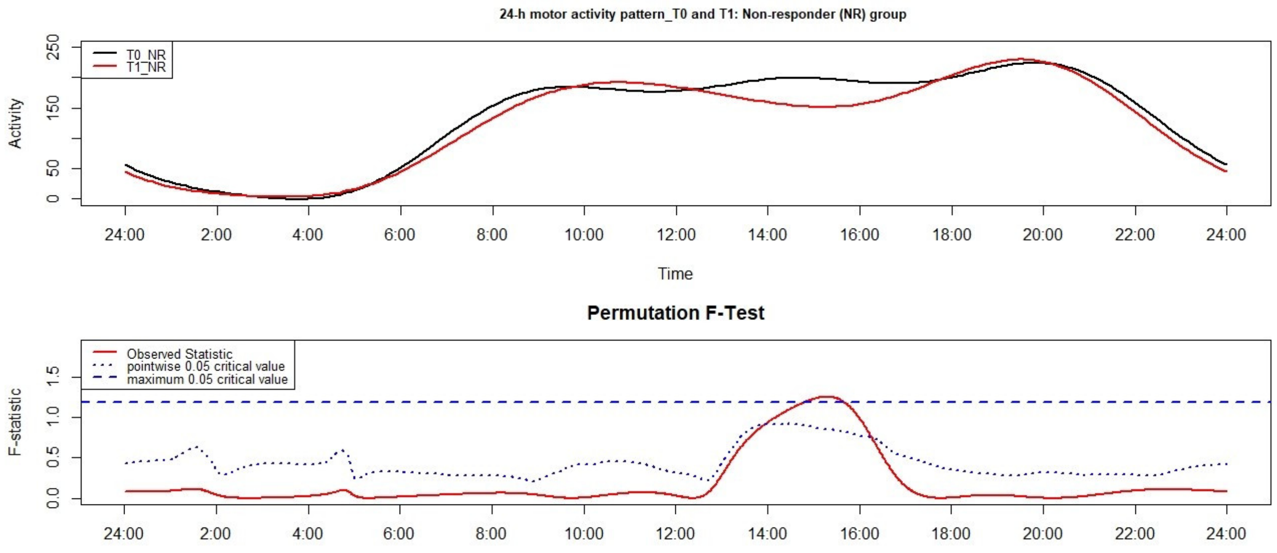

Figure S2 – Results of the functional linear modeling applied to the comparison of 24-h motor activity pattern, monitored at T0 and T1, of the non-responder (NR) group. The functional forms of the 24-h motor activity patterns at T0 and T1 are reported in the upper panel while the results of the non-parametric permutation F-test in the lower panel. Significant results are observed when the solid red line is above the blue dashed line.
